# Serum lactate and phosphate as biomarkers of intestinal ischemia in a Ugandan tertiary hospital: a cross-sectional study

**DOI:** 10.1186/1865-1380-6-44

**Published:** 2013-12-04

**Authors:** Ronald Kintu-Luwaga, Moses Galukande, Francis N Owori

**Affiliations:** 1 Surgery Department, School of Medicine, Makerere University, P.O. Box 7072, Kampala, Uganda

**Keywords:** Bowel ischemia, Biomarkers, Serum lactate, Phosphate

## Abstract

**Background:**

Intestinal ischemia is a common complication of intestinal obstruction and arises from impaired perfusion. The resultant local and systemic inflammatory response and bacterial translocation come with a significant degree of morbidity and mortality. This study therefore aimed to investigate the predictive value of elevated levels of serum lactate and phosphate as biomarkers of intestinal ischemia among patients with mechanical intestinal obstruction.

**Methods:**

This was a cross-sectional analytical study done at Mulago Hospital in Uganda. Ethical approval was obtained. All eligible patients had a blood sample drawn for assay analysis. Determination of bowel ischemia status was by physical examination at laparotomy. Analyses were performed using Stata software, version 10.1, and 2 × 2 tables were used to calculate sensitivity and specificity.

**Results:**

Serum lactate was predictive of bowel ischemia, while phosphate was not. Of the 81 patients enrolled 70 qualified for analysis; 40/70 (57%) had ischemic bowel, while 30/70 (43%) had normal bowel. Among those with ischemic bowel, 28/40 (70%) had reversible ischemia, and 12/40 (30%) had irreversible ischemia. Serum lactate assay had a sensitivity of 66% and specificity of 53% for bowel ischemia in general and a higher sensitivity of 71% and specificity of 80% for irreversible bowel ischemia.

Lactate was predictive of bowel ischemia in general (*p* = 0.011), PPV = 14%, but more significantly predictive of irreversible ischemia (*p* = 0.009), PPV = 42%. NPV for lactate in both forms of ischemia was 93%. Hernias (33/70, 47%) were the most common cause of intestinal obstruction.

**Conclusion:**

Serum lactate assay had moderate sensitivity for bowel ischemia due to acute mechanical intestinal obstruction. The assay can be used to aid diagnosis of bowel ischemia in low technology settings.

## Background

The diagnosis of intestinal ischemia is still a major challenge in sub-Saharan Africa where the acute form of intestinal ischemia caused by intestinal obstruction is most prevalent [1-4]. It is more fatal than the chronic type, therefore requiring early diagnosis and intervention [5,6].

Mechanical intestinal obstruction, the most common cause of bowel ischemia in our setting [1-4], is on the increase. The most common causes of intestinal obstruction are strangulated/obstructed hernia (40-50%), sigmoid volvulus (20%) and adhesions (15-12% of admissions) [3,4]. From recent studies, postoperative adhesions in particular are responsible for up to 40% of patients with intestinal obstruction seen at Mulago Hospital [2]. The most common cause of mechanical bowel obstruction and ischemia in western populations is adhesions.

Clinically useful markers of early gut ischemia would allow for more effective treatment and monitoring, which in turn would reduce the current high morbidity and mortality (10-40%) [1-10]. Efforts to identify an appropriate marker for bowel ischemia have been unsatisfactory and mainly done among western world subjects who have a fairly different spectrum of causes, epidemiology of intestinal ischemia and high level investigative abilities, whereas in resource-limited settings there may be no or only a plain abdominal x-ray as the only available investigation [5-7,10-14]. The epidemiological, genetic and clinical disparities [12-14] are based on the predisposition to causes such as adhesive intestinal obstruction, differences in studied age groups and race-associated genetic differences.

Serological biomarkers such as serum lactate and phosphate have been found useful and for now seem to offer some hope for solving diagnostic challenges associated with intestinal obstruction and ischemia [12-14]. These two markers are released locally when the gut undergoes ischemic injury and increase in blood circulation as the injury advances. They possess a combination of qualities such as ready availability, affordability and user friendliness, and they are minimally invasive. This study therefore sought to determine the diagnostic accuracy of serum lactate and serum phosphate levels in patients with bowel ischemia due to mechanical intestinal obstruction in a resource-limited setting.

## Methods

### Study design

This was a cross-sectional analytical study.

### Study setting

Mulago National Referral Hospital is the main teaching Hospital for Makerere University College of Health Sciences in Kampala. It is situated in Kampala, the capital city of Uganda, and has a 1,500-bed capacity, admitting up to 50 patients daily with various surgical emergencies to its Accident and Emergency Unit.

### Study period

The study was conducted from January to May 2012 inclusive.

### Study population

All patients admitted to the emergency and general surgical wards with a diagnosis of mechanical intestinal obstruction were included.

Patients with potential causes of lactic acidosis, such as diabetic ketoacidosis, severe hypotension, and renal and hepatic failure, were excluded. Patients on antiretroviral drugs, especially stavudine, and those who died or whose symptoms resolved just before surgery, and those younger than 1 year were also excluded.

### Data collection

Data were collected using a questionnaire, including patient demographics and bowel status. Bowel status was categorized as normal bowel, reversibly ischemic and irreversibly ischemic bowel, and serum levels of lactate and phosphate were analyzed. Eligible patients were counseled, consented and recruited by the investigators and assistants during the perioperative period.

### Blood sample handling

Under aseptic techniques, 5 ml of peripheral venous blood was drawn (avoiding the use of a tourniquet), 2.5 ml delivered to a gray-top fluoride container for lactate and 2.5 ml to a plain red-top container for phosphate. Both containers were delivered within 30 min to the laboratory for analysis or stored in a refrigerator in standing position at 4°C in cases when delay in delivery was anticipated. In the laboratory, samples were routinely centrifuged before analysis for 5 min at 10,000 revolutions per minute to obtain serum. The normal laboratory reference serum level ranges were 0.5–2.2 mmol/l for lactate and 0.9–1.5 mmol/l for phosphate (Mulago Hospital Laboratory).

### Scheme for assessment of bowel state

Intraoperatively, the researcher(s) and attending surgeon physically demonstrated and established the absence or presence of ischemia or its sequel by observing gut appearance, texture and the site involved, using clinical judgment that took into consideration the following criteria:

Normal bowel was pink and glistening with normal peristalsis and normal arterial pulsations or mild alteration of one of them.

Reversible ischemic demonstrated a red, dull sheen and reduced peristalsis or arterial pulsations that would improve drastically upon relief of the obstruction.

Irreversible ischemic was dark or black, with a total loss of sheen and permanent reduction or absence of peristalsis and arterial pulsations.

These findings were agreed upon by consensus of the operating surgeon, assistant and investigator(s).

### Quality control

Tourniquet use was always avoided when drawing blood samples. Blood was not drawn from the cannulated limb (with an i.v. line/drip). Late night samples were kept at 4°C in standing position. All samples were centrifuged in the laboratory before analysis.

Random on-the-spot lactate measurements (with a portable lactate meter) were done before samples were delivered to the laboratory to serve as a control for the laboratory measurements. Sample handling was uniform—i.e., transportation and storage were only carried out by the investigator and one research assistant and the handling and processing by one and the same qualified laboratory technologist using the same well-calibrated machine, *Cobas Intergra 400* (Roche), based on an accredited (SANAS) laboratory in Mulago Hospital. Centrifuged samples (serum) were frozen at −20°C in case of the need for sample reruns.

### Data analysis

Analyses were performed using Stata software, version 10.1. Statistical tests included Mann-Whitney U and Fischer’s exact tests. The Kruskal-Wallis equality-of-population rank test was used for independent analysis of factors across three groups defined by bowel status (normal, reversibly ischemic and irreversibly ischemic bowel). Cox regression analysis was used in both univariate and multivariate models. Significant *p* values were ≤0.05. Two-by-two tables were used to calculate the sensitivity and specificity expressed as percentages.

### Ethical considerations

Approval was obtained from the Research and Ethics Committee of Makerere College of Health Sciences, School of Medicine. Informed written consent was obtained from all participants and parental consent for all below 18 years. Those who were incapacitated at the time of data collection were consented later when they were able to consent.

## Results

In this study, 81 patients who fulfilled the criteria were enrolled. Seventy of them had the outcome of interest, and their data were analyzed. Eleven patients were excluded because they had concomitant non-ischemic conditions such as gut perforations, which were not a result of prior obstruction.

Table 1 shows evidence of differences in the distribution of symptoms and physical examination findings.

**Table 1:** Characteristics of patients with bowel ischemia, Uganda study, 2012

**Baseline characteristic**	**N = 70 (100%) total number**	**Normal bowel 30 (43%)**	**Ischemic bowel 40 (57%)**	**p-value (normal vs. ischemic bowel)**
Gender, n (%)				
Female	24 (34)	7 (23)	17 (43)	0.128**
Male	46 (66)	23 (77)	23 (58)	
Age (years), median (IQR)	38 (52–25)	36 (45–25)	41.5 (64–24)	0.251*
Symptom duration, median (IQR)				
Abdominal pain	72 (120–36)	48 (96–24)	96 (14–48)	0.012*
Absolute constipation	48 (96–24)	48 (84–24)	72 (96–48)	0.252*
Relative constipation	24 (48–12)	18 (24–12)	36 (72–20)	0.285*
Abdominal swelling	72 (96–24)	48 (72–24)	72 (120–48)	0.011*
Vomiting	48 (96–24)	24 (96–18)	72 (120–24)	0.103*
Biomarker levels				
Lactate (mmol/l), median (IQR)	1.72 (2.46-1.26)	1.66 (2.1-1.37)	1.77 (2.66-1.21)	0.59*
Phosphate (mmol/l), median (IQR)	1.30 (1.70-1.10)	1.20 (1.5-1.00)	1.40 (1.92-1.10)	0.19*

*Mann-Whitney U test, **Fisher’s exact test. Biomarker values measured by Mulago Hospital Laboratory.

**Figure 1:**
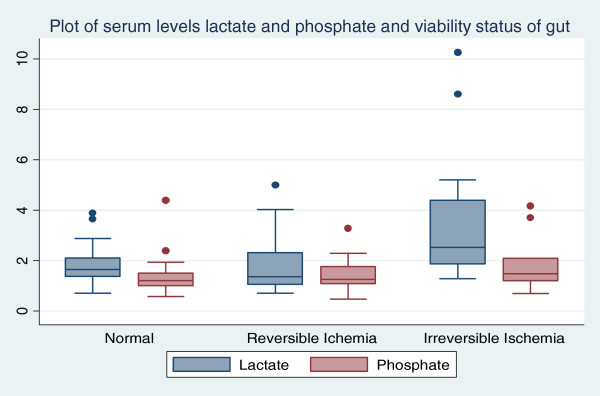
Box plot of serum levels of lactate and phosphate against bowel status.

More males were seen (53; 65%) compared to women (28; 35%), and proportionately more males had both normal and ischemic bowel intraoperatively. The median age group was 38 years (IQR 52-25); however, those with ischemic bowel had a median age of 42 (IQR 64–24), relatively older than those with normal bowel (36 years; IQR 45–25). Patients with bowel ischemia had longer duration of symptoms than those with normal bowel, and the majority of patients with bowel ischemia had most of the signs. There were differences in the distribution of biomarkers. Biomarker levels were higher in patients with bowel ischemia than those with normal bowel as indicated in Figure 1.

A sensitivity of 66% and specificity of 53% for bowel ischemia in general were found, with a positive predictive value of 14% and negative predictive value of 93%. For irreversible ischemia, the sensitivity was 71% and specificity 80%, which correspond to a positive predictive value of 42% and negative predictive value of 93%. Participants with irreversible bowel ischemia had higher levels of serum lactate compared to normal and reversible bowel ischemia groups. There were no major differences in phosphate levels across groups, as shown in Table 2.

**Table 2:** Differences in distribution of patient clinical factors by bowel status

**Characteristic/predictor**	**X**^**2**^**(Df)**	p-value*
Gender	7.135 (2)	0.028
Age	2.820 (2)	0.244
Symptoms duration (hours)		
Abdominal pain	6.450 (2)	0.040
Absolute constipation	1.318 (2)	0.517
Relative constipation	1.894 (2)	0.388
Abdominal swelling	6.556 (2)	0.038
Vomiting	2.723 (2)	0.256
Radiological findings		
Air in pelvis	2.723 (2)	0.256
Biomarkers		
Lactate levels	9.090 (2)	0.011
Phosphate levels	3.470 (2)	0.176

*Kruskal-Wallis equality-of-populations rank test.

With the *Kruskal-Wallis equality-of-population rank test*, factors were compared and analyzed independently among three groups defined by bowel status (normal bowel vs. reversibly ischemic bowel vs. irreversibly ischemic bowel). Significant factors were gender (0.03), abdominal pain duration (0.04), abdominal swelling duration (0.04) and lactate levels (0.01), as indicated in Table 3.

**Table 3:** **Univariate and multivariate assessment of factors associated with bowel status across two groups,** n***ormal*vs. *irreversible ischemic bowel, *using modified cox regression models for cross-sectional studies**

	**Univariate**			**Multivariate**		
**Characteristic**	**PRR**	**[95%] CI**	**P value**	**PRR**	**[95%] CI**	**P value**
Gender: male vs. female	0.264	0.080 - 0.877	0.030	0.251	0.038 - 1.667	0.152
Age	1.019	0.990 - 1.049	0.200	1.028	0.988 - 1.069	0.168
Symptom duration (h)						
Abdominal pain	1.000	0.994 - 1.007	0.928	-	-	-
Absolute constipation	1.002	0.991 - 1.012	0.770			
Relative constipation	1.037	0.972 - 1.105	0.272			
Abdominal swelling	1.002	0.993 - 1.009	0.677			
Vomiting	1.002	0.991 - 1.014	0.688			
Signs						
Abdominal distension	0	-	-	-	-	-
Tenderness	0.961	0	1.000			
Bowel sounds	-	-	-			
Degree of dehydration	-	0.124 - 7.444	0.970			
Radiological findings						
Bowel loop distension	-	-	1.000	-	-	-
Air-fluid levels	0	0	0.238			
Air in pelvis	3.500	0.438 - 27.984	-			
Biomarkers						
Lactate levels	1.357	1.311 - 2.919	0.001	1.391	1.086 - 1.781	0.009
Phosphate levels	1.437	0.865 - 2.388	0.162	0.981	0.464 - 2.074	0.960

*PRR* = Prevalence rate ratios.

Associations with irreversible bowel ischemia were with gender (*p*-value, 0.03), while lactate had a persistently significant *p*-value (0.001 and 0.009) in both univariate and multivariate models, respectively. Phosphate levels were not significant in both analyses and models, indicating very low sensitivity and specificity (see Table 3). Sensitivity and specificity of the significant lactate assay were calculated using a cutoff 1.5 times the upper normal range of lactate (2.2 mmol/l) in the two-by-two tables.

The majority of patients had normal bowel (30, 43%), but they were slightly more than the reversible ischemia group, which had 28 (40%). For irreversible ischemia (12/40), gangrene was the most common feature; however, several patients had a mixed pattern of infarction, gangrene and necrosis.

The ileum was the main part of the gut involved (52, 72%), followed by the colon (27, 38%), and the omentum (6, 8.3%) was the least. For all gut parts, there were comparatively more patients with ischemic bowel than normal bowel. Groin hernias were the main cause of intestinal obstruction (47%), followed by gut volvulus (23%), then adhesions (17%) and intussusceptions (14%).

There was a weak correlation between lactate level and age and a weak negative correlation between phosphate level and age. There was no difference in the distribution of serum levels of lactate and phosphate by gender. There was no correlation of duration of symptoms and serum levels of both lactate and phosphate.

Costs per test were US$ 2 and US$ 3 for lactate and phosphate assays, respectively.

## Discussion

We set out to investigate the diagnostic value of the association between serum lactate and phosphate in relation to bowel ischemia. There was an association between serum lactate and bowel ischemia, but not between serum phosphate and bowel ischemia. The population studied was mostly male, with a male:female ratio of 2:1, though the bioassays didn’t vary by gender. The mean age was 39 years; this would be considered young in the context of mechanical intestinal obstruction.

### Role of biomarkers

The role of serum lactate in predicting bowel ischemia was modest; it was far below the higher values of sensitivity found by several studies [12,15]. However, the study by Lange et al. [12] did not specify the degree of ischemia. For irreversible ischemia, which includes infarcted gut, the sensitivity and specificity were much higher, 71% and 80%, respectively, in this study. This is close to the findings of Klein et al. where serum lactate levels were elevated in 77% of all patients with bowel infarction [16].

Gearhart et al. found 100% sensitivity for intestinal ischemia with a combination of alpha-glutathione S-transferase and lactate [17]. This high degree of sensitivity may also be attributed to the use of more rigorous inclusion criteria such as the use of a combination of colonoscopy, angiography and autopsy to confirm the diagnosis of bowel ischemia. In this study, we only relied on physical inspection to determine bowel status. The specificity of 42% by Lange is however closer to the 53% found in this study.

Phosphate had no association with bowel ischemia in this study, which is a departure from the findings of Jamieson [18], who found phosphate to be significantly increased (in 80% of patients) in massive bowel ischemia. He however did not define what massive was.

Both biomarker levels were higher in patients with bowel ischemia than in those with normal bowel, implying that rising serum levels of lactate and phosphate may predict bowel ischemia. Although the overall change in the two biomarker levels was low, it was more significant for lactate (*p* = 0.009). For phosphate, it was insignificant (*p* = 0.960). The weak correlation with phosphate was further confirmed (see Table 3), where the *p* value was > 0.05. These findings are comparable to those by Leo [19] where phosphate sensitivity was as low as 26%. The differences in these values ought to be regarded with due consideration of the clinical, demographic and geographical disparities and their impact on human physiology, for instance, with an average symptom duration of 72 h or 3 days, it is possible that phosphate may have been cleared in the urine [13].

### Bowel ischaemia

The majority of patients had ischemia 40/70 (57%). The median duration of abdominal pain was 72 h (IQR 120-36) (*p* = 0.012); long symptom duration is considered a risk factor for bowel ischemia [2,4]. Symptom duration had a correlation with intraoperative findings, specifically abdominal pain and swelling. Most patients with bowel ischemia had the complete symptomatology and had had symptoms for a longer period of time than those with normal bowel.

Ileum was the most common part of the gut involved in the obstruction (52%), mainly contained in obstructed/incarcerated hernias, which was also the most prevalent intraoperative diagnosis. These findings correspond rather accurately with those of Kiyengo [20] where the incidence of ileum to colon involvement was at a 2:1 ratio. In some cases, more than one part of the gut or accessory organs would be entrapped in hernias or intestinal volvulus, commonly the omentum, peritoneal fat and appendix or jejunum-ileum and ileum-colon, respectively. The cecum and appendix were mainly components of intussusceptions. All these parts have been commonly involved in the various forms of mechanical intestinal obstruction recorded before in other studies in Uganda and other parts of sub-Saharan Africa [1-4,8], whereas a definitive diagnosis of mechanical obstruction with features of bowel ischemia warrants a laparotomy in situations of adhesive bowel obstruction; nonoperative measures are employed first though within time limits of 12–36 h.

In this study the main causes of mechanical intestinal obstruction were hernias (33%). The reason for the prevalence of intestinal obstruction caused by hernias is that many patients live with untreated hernias as opposed to western countries where access to care is early. Other causes included sigmoid volvulus (14%), adhesions and intussusceptions in declining order of proportion. These study findings are similar to those of MacAdam (1960) [1] and Watya (1992) [8], who registered hernias as the leading cause at 54%, sigmoid volvulus at 21% and adhesive obstruction at 9%. This pattern however is different from the one reported by Kimuli (2006) [2], in which adhesions were 40%, followed by sigmoid volvulus at 32%, tumors at 12%, intussusceptions 7% and fecal impaction 7%.

In western countries, mechanical intestinal obstruction (and the resultant intestinal ischemia) is mainly caused by post-surgical adhesions in over 60% of cases [21,22].

### Study limitations

Though serum lactate is non-specific for bowel ischemia, it may be useful in settings where high-level investigative abilities are limited. The physical assessment of bowel status has limited accuracy (70–89%) [23,24]. The ideal assessment for bowel ischemia should be histological (requiring biopsy), which allows assessment of the entire gut wall including mucosa where the ischemia starts; this wasn’t feasible though. Some patients with apparently normal or viable bowel could have had raised levels of serum lactate and phosphate, i.e., ischemia may have been present in the inner gut layers; type I ischemia is not appreciable physically. Therefore, this limited this study to type 2 and 3 bowel ischemia.

Patients with mechanical intestinal obstruction are operated on as soon as they are resuscitated, but sometimes logistical limitations lead to delays. Whereas the majority of patients did not wait longer than 6 h, it is possible that during this waiting time significant changes in bowel status and biomarker levels occurred. From the single blood sample drawn, it was not possible to observe the ongoing changes in biomarker levels. Large-scale studies are needed especially in sub-Saharan Africa to further explore the role of serum lactate and phosphate assays in aiding bowel ischemia diagnosis.

## Conclusion

Serum lactate assay had moderate sensitivity for bowel ischemia caused by acute mechanical intestinal obstruction in a sub-Saharan Africa resource-limited setting. This assay may augment the diagnosis of bowel ischemia in the emergency setting.

## Abbreviations

I.V: Intravenous; IQR: Interquartile ratio; NPV: Negative predictive value; PPV: Positive predictive value.

## Competing interests

The authors declare that they have no competing interests.

## Authors’ contributions

GM conceived the concept. KLR collected the data, participated in the analysis and wrote the first draft. GM and OFN contributed to revising the drafts. GM performed critical reviews of the manuscript. All authors approved the final manuscript.
